# The UF Deep Brain Stimulation Cognitive Rating Scale (DBS-CRS): Clinical Decision Making, Validity, and Outcomes

**DOI:** 10.3389/fnhum.2020.578216

**Published:** 2020-09-29

**Authors:** Lauren Kenney, Brittany Rohl, Francesca V. Lopez, Jacob A. Lafo, Charles Jacobson, Michael S. Okun, Kelly D. Foote, Dawn Bowers

**Affiliations:** ^1^Department of Clinical and Health Psychology, College of Public Health and Health Professions, University of Florida, Gainesville, FL, United States; ^2^Norman Fixel Institute for Neurological Diseases, University of Florida, Gainesville, FL, United States

**Keywords:** Parkinson’s disease, deep brain stimulation, cognition, neuropsychology, scale validation

## Abstract

To more efficiently communicate the results of neuropsychological assessment to interdisciplinary teams, the University of Florida Neuropsychology Service developed a Deep Brain Stimulation-Cognitive Rating Scale (DBS-CRS). This tool condensed results of a 3-h exam into a five-point scale ranging from 1 (least) to 5 (most) cognitive concern for DBS surgery. In this study, we evaluated the role of the DBS-CRS in clinical decisions by the interdisciplinary team to proceed to surgery, its relationship to objective neuropsychological scores, and its predictive utility for clinical outcome. We retrospectively examined 189 patients with Parkinson’s disease who were evaluated for DBS candidacy (mean age 64.8 [*SD* 9.2], disease duration 8.9 years [*SD* 5.0], UPDRS-Part III off medication 38.5 [*SD* 10.5], Dementia Rating Scale-II 135.4 [*SD* 6.0]). Approximately 19% of patients did not proceed to surgery, with neuropsychological red flags being the most commonly documented reason (57%). Patients who underwent DBS surgery had significantly better DBS-CRS scores than those who did not (*p* < 0.001). The two strongest and unique neuropsychological contributors to DBS-CRS ratings were delayed memory and executive function, followed by language and visuoperception, based on hierarchical linear regression that accounted for 77.2% of the variance. In terms of outcome, DBS-CRS scores were associated with higher quality of life, less severe motor symptoms, and better daily functioning 6 months following DBS surgery. Together, these findings support the construct and predictive validity of the DBS-CRS as a concise tool for effectively communicating pre-DBS cognitive concerns to an interdisciplinary team, thereby aiding decision making in potential DBS candidates.

## Introduction

Deep brain stimulation (DBS) is a powerful treatment which can be used for many of the motor symptoms, and also the motor fluctuations, associated with Parkinson’s disease (PD). DBS involves the implantation of electrical leads deep into the brain and can be applied to one of several target sites—typically the subthalamic nucleus (STN) or the globus pallidus internus (GPi); the DBS leads deliver high-frequency stimulation and are controlled by a pulse generator usually implanted in the chest wall. Since approval by the U.S. Food and Drug Administration in 1997, more than 150,000 DBS surgeries have been performed, and careful patient selection has emerged as a major determinant for clinical success (Wagle Shukla and Okun, 2016). Selection involves precise diagnosis by fellowship-trained movement disorders neurologists, an assessment of medication response, an interdisciplinary screening, optimal pre-surgical medication management, and formal neuropsychological testing (Okun et al., 2005). Potential DBS candidates are screened for accurate diagnosis, functional disability, and duration, severity, and progression of motor and non-motor symptoms. Many centers follow at least a minimal interdisciplinary team approach, considering neurological, neurosurgical, psychiatric, occupational/physical therapy, and neuropsychological assessment (Abboud et al., 2014).

A major goal of pre-DBS neuropsychological testing is to provide the patient and the treatment team with information pertinent to the risk-benefit ratio of pursuing surgery; this includes whether patients can successfully meet pre- and post-operative demands (i.e., informed consent, medication and device adherence, coping with stress). It also includes the identification of any cognitive or psychological contraindications to DBS (i.e., dementia or cognitive profiles atypical for PD, thus suggestive of another disease) (Tröster, 2017). To address these questions, the interpretation of neuropsychological test scores must be individualized to each patient and relies on clinical judgment to place scores in the context of factors such as age, ethnicity, disease duration, educational and occupational attainment, mood, motivation, and situational factors arising during testing. Further, the neuropsychologist will consider qualitative aspects not typically captured by traditional scoring methods (i.e., the patient’s approach to a task). While complex and nuanced formulation of the neuropsychologist’s conclusions are important to document, a recent nationwide survey revealed that most referral sources pay the greatest attention to diagnosis and to recommendations (Postal et al., 2018). Thus, there is a need for a simplified method to convey a concise summary of DBS screening related neuropsychological findings.

In order to communicate the results of the pre-DBS neuropsychological evaluation more efficiently to an interdisciplinary team, the neuropsychology service at the University of Florida (UF) created a Likert rating scale—the UF Deep Brain Stimulation Cognitive Rating Scale (DBS-CRS)—which ranges from 1 (least) to 5 (most) cognitive concern for surgery. Although the scale has been used at UF clinically since 2013, the relationship of DBS-CRS scores to the decision to proceed to surgery and to objective neuropsychological test scores had not been previously examined. Important questions remain such as whether the recommendations communicated via the DBS-CRS had an effect on the decision to proceed to surgery. Other questions also emerged including what patterns of neuropsychological performance drive clinical decision making, thereby leading to higher (worse) scores on the DBS-CRS and thus potentially raising a red flag for DBS cognitive risk. Finally, we were interested in whether scores on the DBS-CRS were predictive of quality of life post-DBS surgery.

The current study had three aims. First, we wanted to determine how recommendations communicated via the DBS-CRS impacted the decision to proceed to surgery by the interdisciplinary team. We hypothesized that cognitive concerns would emerge as the most common reason for a recommendation against DBS surgery. In these cases, we predicted that higher (worse) DBS-CRS scores would be present. This hypothesis was based on the assumption that other common reasons for not proceeding with DBS surgery (i.e., unusually low response to levodopa, psychogenic/overlay symptoms, unstable psychiatric symptoms, etc.) would have been informally identified and excluded prior to a formal interdisciplinary evaluation for DBS candidacy.

Second, we sought to learn what aspects of the objective neuropsychological examination raised concerns for cognitive risk and thus led to worse scores on the DBS-CRS. In PD, a ‘fronto-executive’ profile on neurocognitive testing is a common result (Zgaljardic et al., 2003), and this result corresponds to underlying disease pathology and subtle deficits. These subtle deficits would not be viewed as a worrisome risk factor for DBS surgery. What would serve as a ‘red flag’ would be more blatant atypical profiles (e.g., primary progressive aphasia, memory retention and naming deficits suggestive of Alzheimer’s syndrome) or profiles that reflected more pervasive and severe neuropsychological deficits (executive, memory, and otherwise), thus leading to worse ratings. To address this question, we performed regression analyses to examine the contribution of neuropsychological domains to the DBS-CRS score and multivariate analysis of variance to examine the cognitive patterns across DBS-CRS scores.

Third, we examined whether scores on the DBS-CRS were associated with self-reported quality of life during the 6-month period following DBS surgery. We focused on quality of life, as it is closest to a patient centric perspective for this analysis, rather than focusing on strict cognitive outcomes (Floden et al., 2015). We hypothesized that better scores on the DBS-CRS would be associated with improved scores on a validated quality of life measure, the Parkinson’s Disease Questionnaire-39 (PDQ-39; Jenkinson et al., 1997). Finally, we also examined mood/motivation scores (Beck Depression Inventory II; Beck et al., 1996; Apathy Scale; Starkstein et al., 1992) and the motor and activities of daily living (ADL) scores derived from the Unified Parkinson’s Disease Rating Scale (UPDRS; Fahn et al., 1987).

## Method

### Design

**FIGURE 1:**
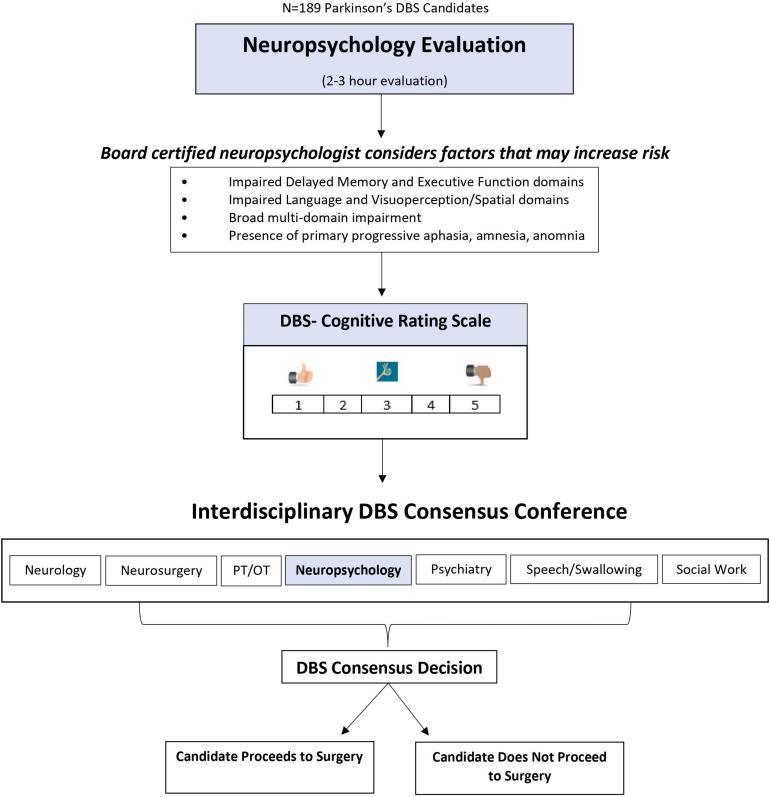
Overview of Neuropsychology’s use of the DBS-CRS in deep brain stimulation surgery candidate selection.

This study involved a retrospective chart review of individuals with PD who were potential DBS surgery candidates at the UF Norman Fixel Institute for Neurological Diseases. Data encompassed information prior to potential DBS surgery (i.e., demographic information, motor symptom severity and ADL scales, medication use, neuropsychological and mood/motivation measure scores, DBS consensus conference meeting notes, and subsequent DBS surgery notes). Six-month follow-up included quality of life, mood/motivation, and motor scales. Study procedures were approved by the University of Florida Institutional Review Board. Figure 1 depicts the overall study flow of participants through the various procedures, which are described in detail in the following sections.

### Participants

Participants included 189 patients being considered for DBS surgery between 2013 and 2018. This time range was based on when the DBS-CRS began to be used (2013) and an arbitrary stop point of 2018 for data collection. Referral for DBS surgery was generally contingent on motor symptom responsivity to levodopa (typically 30–40% reduction) and absence of conspicuous dementia. All DBS candidates underwent a 2-day “Fast Track” evaluation consisting of independent evaluations by specialists in the following areas: neurology, neurosurgery, neuropsychology, psychiatry, physical and occupational therapy, speech and swallowing therapy, and social work.

To be included, participants had a diagnosis of PD, as determined by UF movement disorders neurologists, and were a candidate for first-time DBS surgery. Exclusion criteria included: (a) history of previous neurosurgery; (b) diagnosis of any additional or comorbid movement disorders (i.e., dystonia or combined PD-essential tremor diagnoses) as determined by UF movement disorders neurologists. For follow-up analyses, patients were excluded if they experienced an intra-operative adverse event (e.g., stroke), post-operative infection, or had bilateral DBS surgery.

### Neuropsychological, Mood, and Quality of Life Measures

All DBS candidates completed a battery of neuropsychological, mood, and motivation measures that took between 2 and 3 h to complete. The battery consisted of a cognitive screening measure, the Dementia Rating Scale -2 (DRS-2; Jurica et al., 2001), and standard neuropsychological measures of attention/working memory, delayed recent memory, language, visuoperception, and executive function. Specific tests are shown in Table 1 and are grouped by cognitive domain based on theoretical considerations (Kirsch-Darrow, 2010; Jones et al., 2014, 2017). Norms for each test were derived from test-specific manuals or previously published norms (Heaton et al., 2004) and then converted to z-scores. For each cognitive domain (e.g., memory, executive function, etc.), a composite score was computed by averaging individual z-scores of tests within a domain to create a domain-specific composite. We did not include the DRS-2 within our domain composites—instead, using it as a general index of cognitive impairment.

**TABLE 1:** Neuropsychological tests within each cognitive domain composite.

Cognitive domain	Tests	Raw score used
**Delayed Recent Memory**	HVLT-R WMS-III Logical Memory	Delayed Total Recall Delayed Total Recall
**Executive Functioning**	Stroop Test (Interference Trial) TMT Part B Letter Fluency (FAS)	Total Number of Correct Items Completion Time Total Number of Words (All Three Trials)
**Language**	BNT Semantic Fluency (Animals)	Total Correct Spontaneous Responses Total Number of Words
**Visuoperceptual Functioning**	Benton JOLO Benton FRT	Total Items Correct Total Items Correct
**Attention/Working Memory**	WAIS-III Digit Span Forward WAIS-III Digit Span Backward	Total Number of Points Total Number of Points

*WMS-III, Weschler Memory Scale-Version III (Wechsler, 1997b); HVLT-R, Hopkin’s Verbal Learning Test-Revised (Brandt, 1991); Stroop Test is the Golden version (Golden, 1978); TMT Part B, Trails Making Test Part B (Army Individual Test Battery, 1944); Letter Fluency (FAS) (Spreen and Benton, 1977); BNT, Boston Naming Test (Kaplan et al., 1983); Semantic Fluency (Animals) (Goodglass and Kaplan, 1983); Benton JOLO, Benton Judgment of Line Orientation (Benton et al., 1978); Benton FRT, Facial Recognition Test (Benton et al., 1994); WAIS-III, Weschler Adult Intelligence Scale-Version III (Wechsler, 1997a).*

*Mood* and motivation measures included the Beck Depression Inventory II (BDI-II; Beck et al., 1996), the Apathy Scale (AS; Starkstein et al., 1992), and the State-Trait Anxiety Inventory (STAI; Spielberger et al., 1983). Participants also completed a health-specific *quality of life scale*, the Parkinson’s Disease Questionnaire -39 (PDQ-39; Jenkinson et al., 1997; Martinez-Martin et al., 2011). Additionally, we obtained Part II scores from the UPDRS (ranging from 0 to 52), an indicator of patient-reported activities of daily life, as another proxy for quality of life. *Motor disease severity* was indexed by the motor exam from the UPDRS Part III (ranging from 0 to 72). On these measures, higher scores indicated greater severity of symptoms and worse quality of life.

### UF Deep Brain Stimulation – Cognitive Rating Scale (DBS-CRS)

Patients were assigned a cognitive risk rating based on the neuropsychological findings by a board-certified neuropsychologist (DB) in conjunction with neuropsychology trainees. A five-point Likert scale, known as the Deep Brain Stimulation- Cognitive Rating Scale (DBS-CRS), was used to clearly and simply convey concerns, if any, about the potential cognitive risks for moving forward with DBS surgery. The rating guidelines included the following.

(1) Scores of 1 were reserved for individuals who were deemed “cognitive superstars,” with no areas of cognitive impairment.
(2) Scores of 2 reflected minimal cognitive weaknesses that were isolated to the executive domain.
(3) Scores of 3 were given to individuals with more pronounced executive impairments, but consistent with cognitive sequelae of PD.
(4) Scores of 4 reflected cognitive impairment, worse than expected for PD, or atypical impairments (i.e., global memory difficulties) that might be suggestive of other disease entities (i.e., amnestic Mild Cognitive Impairment).
(5) Scores of 5 indicated severe cognitive impairment, including PD dementia, or other cognitive patterns suggestive of other diagnoses (e.g., primary progressive aphasia). Surgery was not recommended.

Regarding memory, poor performance on word list learning tasks was viewed as a more frontally mediated deficit and part of the executive profile based on a robust literature with non-demented PD and older adults (Tremont et al., 2000; Zahodne et al., 2011). However, co-occurrence of impaired recent memory for semantically meaningful information, like novel stories, was viewed as prognostic of a true amnestic profile.

In summary, these DBS-CRS ratings were based on clinical judgment and interpretation of the neuropsychological exam. They served to distil and communicate recommendations by neuropsychology to the interdisciplinary team.

### Interdisciplinary Consensus Conference

Each DBS surgery candidate was discussed at an interdisciplinary monthly conference, where the team arrived at a consensus decision regarding recommendations for DBS surgery. Reasons for not proceeding with surgery were coded in the following categories: Cognitive concerns only, Cognitive concerns plus another concern, Neurological concerns only, Psychiatric concerns only, General health concerns only, or Multiple reasons (excluding cognitive concerns). Confirmation of DBS surgery was based on medical chart review. Patients who were cleared for DBS surgery but later decided not to proceed were coded as “Patient choice.” The reasons for not proceeding ranged from ‘fears’ about being constrained by the head ring (i.e., claustrophobia) to improved symptoms following dopaminergic medication optimization.

### Statistical Analysis

SPSS Version 25 (IBM, 2017) was used to conduct all the following analyses. Due to the clinical nature of our data, not all participants completed all measures. Thus, we used pairwise exclusion criteria for the analyses. Exact analyses used are specified within the results.

## Results

### Sample Characteristics

As shown in Table 2, the final sample of 189 Parkinson’s patients ranged in age from 38 to 83, with an average age of 64.8 years. As a group, the participants were well-educated, predominantly male (72.5%), and Caucasian (93.7%), and had an almost 9-year duration of a PD diagnosis. Scores on UPDRS motor exam (III) reflected moderate disease severity when tested off dopamine medications. This score improved by 36% when tested “on medication,” a general indicator of potentially good responsivity of PD symptoms to DBS surgery (Hartmann et al., 2019). As a group, scores on indices of depression (BDI-II), apathy (AS), and anxiety (STAI) were below clinical cutoff, though there was substantial variability across participants. Approximately 37% of the sample were taking antidepressant medications, and 36.5% were taking anxiolytic medications.

**TABLE 2:** Sample characteristics and comparisons between those who proceeded to surgery (DBS+) versus those who did not (DBS−).

Measure	Overall sample		DBS + (*n* = 154)		DBS− (*n* = 35)		Significance testing (DBS+ vs. DBS−)	
*Variable*	*Mean/%*	*SD*	*Mean/%*	*SD*	*Mean/%*	*SD*	*Z Statistic^∧^*	*p-Value*
Age	64.79	9.15	64.32	9.01	66.91	9.88	−1.71	0.088
Education (years)	14.94	2.63	14.95	2.61	14.89	2.77	0.32	0.753
Years Since Diagnosis	8.90	4.98	8.65	4.66	10.01	6.17	−1.01	0.312
% Male^1^	72.5%		70.8%		80%			0.512
% Caucasian^1^	93.7%		94.8%		88.6%			0.601
UPDRS III, on medication	24.92	10.27	23.91	9.62	29.45	11.91	−2.71	0.007*
UPDRS III, off medication	38.53	10.54	37.74	9.84	42.00	12.74	−2.07	0.039*
% Change in UPDRS from off to on meds	−35.9%	18.54	−37.2	18.27	−30.0	18.89	1.73	0.083
% Taking Antidepressant Medication^1^	37.0%		36.4		40.0%			0.688
% Taking Antianxiety Medication^1^	36.5%		39.0%		25.7%			0.142
BDI-II, raw total	10.29	7.03	9.97	6.63	11.76	8.62	−0.77	0.444
STAI: State Anxiety, percentile	66.91	30.07	65.81	29.72	72.16	31.67	−1.39	0.164
STAI: Trait Anxiety, percentile	61.96	30.85	60.05	30.75	71.19	30.18	−1.89	0.059
Apathy Scale, raw total	11.84	5.94	11.83	6.03	11.91	5.56	−0.17	0.864
**DBS-Cognitive Rating Scale** (1 to 5)	2.69	1.00	2.47	0.89	3.66	0.90	−5.88	<0.001*
Dementia Rating Scale-2, raw total Cognitive Composites (z-scores)^#^	135.36	6.04	136.40	4.83	130.62	8.41	4.29	<0.001*
Delayed Memory	−0.38	1.07	−0.21	1.04	−1.15	0.89	4.92	<0.001*
Executive Functioning	−0.61	0.90	−0.49	0.83	−1.20	1.00	3.74	<0.001*
Language	−0.07	0.95	0.05	0.92	−0.60	0.92	3.65	<0.001*
Visuoperceptual Functioning	0.13	0.77	0.17	0.78	−0.10	0.74	1.79	0.073
Attention/Working Memory	0.15	0.82	0.22	0.83	−0.19	0.68	2.48	0.013*

UPDRS, Unified Parkinson’s Disease Rating Scale; BDI-II, Beck Depression Inventory -II; STAI, State-Trait Anxiety Inventory. ^#^z-score has a mean of 0 and SD of 1; z-score composites computed from performance on neuropsychological tasks within a domain. ^∧^Used Mann–Whitney *U*-test to determine significant group differences. ^1^Used Pearson chi-square goodness of fit to determine significance group differences. *Difference was significant at alpha = 0.05.

The DBS-CRS ratings across the PD patients ranged from 1 to 5, with a mean of 2.69 (*SD* = 1.00) and were slightly kurtotic in distribution (z-kurtosis = −1.92, *p* < 0.03; Kolmogorov–Smirnov, *p* < 0.001). Examining the relationship of DBS-CRS scores and other PD disease characteristics revealed that DBS-CRS scores did not significantly correspond with years since diagnosis (*p* > 0.05), but they did correspond to motor symptom severity whether on or off medications (UPDRS Part III; *r*’s = 0.26, *p*’s < 0.001). As a group, performance on a dementia screener (DRS-2) was above the clinical cutoff, though scores ranged from 100 (impaired) to 144 (maximum possible). Scores on individual cognitive composites from the neuropsychological exam indicated worst performance in the executive domain, which is typical for individuals with PD. Results of a repeated measures analysis of variance [ANOVA; *F*(3.60,442.63) = 25.54, *p* < 0.001] and Bonferroni corrected pairwise comparisons reflected the following pattern of significant findings (p’s < 0.05): Executive Function < Delayed Memory < (Language = Visuoperceptual = Attention/Working Memory).

### The DBS-CRS and Proceeding to DBS Surgery

Of the 189 patients who underwent DBS Fast Track evaluation, 35 (18.5%) did not proceed to DBS surgery. The most common reason was cognitive concern (57%) and included 10 individuals for whom cognition was the sole reason and another 10 for whom cognition was one of multiple reasons. The next most common reason for not proceeding with DBS surgery was patient choice (23%) because of reasons such as claustrophobia (related to the head rim used during surgery) or adequate response to dopaminergic medications. Other patients did not proceed due to psychiatric concerns only (9%), multiple reasons (excluding cognitive concern; 6%), general health concerns (3%), and neurological concerns (3%).

Comparison of characteristics of patients who did and did not proceed to DBS surgery is shown in Table 2. Patients who proceeded to DBS surgery (DBS+) had significantly better (i.e., lower) DBS-CRS scores than those who did not (DBS−). Indeed, 85% of the patients who did not proceed with DBS surgery due to cognitive concerns (alone or along with other reasons) received DBS-CRS scores of 4 or 5, with the remainder receiving scores greater than 3.

Further comparisons of the DBS+ and DBS− groups indicated that the two groups differed on motor (UPDRS on and off) and cognitive symptoms. Motor-wise, scores on the UPDRS-III, both on and off medication, were significantly better for those who underwent surgery (DBS+) relative to those who did not (DBS−). Cognitively, patients who had surgery (DBS+), relative to those who did not (DBS−), scored significantly better on the DRS-2 and all neuropsychological domain composites, except visuoperception. In contrast, the DBS+ and DBS− groups were similar across age, education, sex distribution, disease duration, mood scales, and use of anti-depressant and anti-anxiety medications.

### Clinical Decision Making (DBS-CRS): Contribution of Neuropsychological Domains to DBS-CRS Scores

To identify what domains of the neuropsychological exam contributed to the DBS-CRS ratings, we performed a hierarchical linear regression. The first block in the model included ‘non-cognitive’ variables that were significantly correlated with DBS-CRS ratings, based on Spearman rho analyses. These variables included education and scores on the BDI-II, AS, and the STAI (both trait and state scores) and can be conceptualized as ‘covariates.’ Block 2 included the five cognitive domain composite scores: Executive Function, Delayed Memory, Attention/Working Memory, Language, and Visuoperception.

Regression results indicated an overall significant model [*F*(10,111) = 37.65, *p* < 0.001] that accounted for 77.2% of the variance. The first block of education and mood scores significantly predicted DBS-CRS scores [*F*(5,116) = 2.59, *p* = 0.029] and accounted for 10.1% of the variance. Adding the second block of cognitive composites significantly improved the model [Δ*R*^2^ = 0.67, Δ*F*(5,111) = 65.6, *p* < 0.001]. After bootstrapping, due to non-normality of the data, the relative strengths of the individual cognitive domain predictors of the DBS-CRS scores were as follows: Delayed Memory (β = −0.44, *p* = 0.001), Executive Function (β = −0.39, *p* = 0.001), Language (β = −0.14, *p* = 0.023), and Visuoperception (β = −0.12, *p* = 0.021). The only domain that was not significant was Attention/Working Memory (β = 0.06, *p* = 0.223). Thus, the two strongest and unique contributors to the DBS-CRS scores were performance on delayed recent memory and executive function tasks.

**FIGURE 2:**
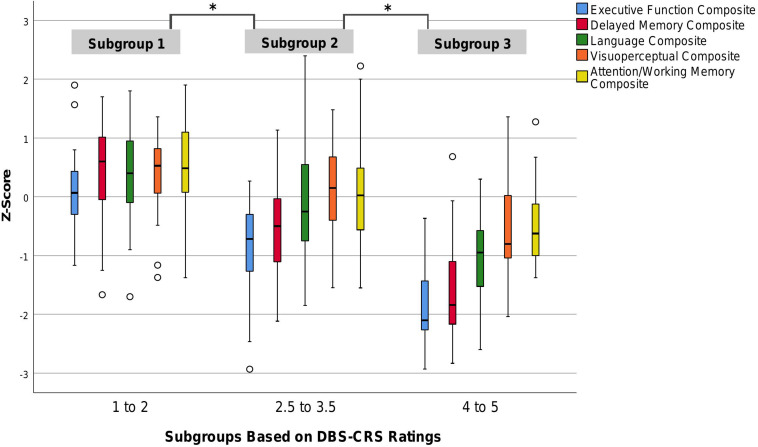
Performance on domain-specific cognitive composites by patients in three DBS-CRS subgroups: Strong (1–2), Average-Expected (2.5–3.5), Poor (4–5). z-score for composite has mean of 0, SD of 1; circles represent outliers; ^∗^*p* < 0.005 difference among each subgroup (Executive Function (*F*[2,121] = 59.40, *p* < 0.001); Delayed Memory (*F*[2,121] = 47.87, *p* < 0.001); Language (*F*[2,121] = 16.51, *p* < 0.001); Visuoperception (*F*[2,121] = 18.46, *p* < 0.001); Attention/Working Memory (*F*[2, 121] = 5.81, *p* = 0.004)); Bonferroni corrected comparison of domain-specific cognitive composite across subgroups: Executive Function, Delayed Memory, Language, and Visuoperception (group 1 > group 2 > group 3, all *p*’s < 0.05); Attention/Working Memory (group 1 < [group 2 = group 3], *p*’s < 0.05, *p* > 0.05, respectively).

A subsequent multivariate analysis of variance (MANOVA) examined the pattern of the neuropsychological domain scores associated with the DBS-CRS ratings. To maximize interpretative clarity, we created three Subgroups based on DBS-CRS ratings: (a) those with relatively good ratings (*N* = 67, ratings 1 to 2), (b) those with more average ratings (*N* = 91, ratings of 2.5 to 3.5), and (c) those with poor ratings (*N* = 31, ratings of 4 to 5). Figure 2 depicts neuropsychological domain scores for these three subgroups. Results of the MANOVA were significant [Hotelling’s trace, *F*(10,232) = 19.17, *p* < 0.001], as were subsequent *post hoc* analyses (univariate and Bonferroni *t*-tests). Neuropsychological performance was significantly better as a function of DBS-CRS ratings, with Subgroup 1 > Subgroup 2 > Subgroup 3. This pattern was present for four of the cognitive domains (Executive Function, Delayed Memory, Language, Visuoperception) based on *post hoc* comparisons. For the Attention/Working Memory domain, performance was as follows: Subgroup 1 > (Subgroup 2 = Subgroup 3). These findings align with view that poorer DBS-CRS ratings are associated with worse cognitive performance across neuropsychological domains.

### DBS-CRS and DBS Outcome: Quality of Life, Mood, and UPDRS

We sought to uncover whether ratings based on the DBS-CRS were associated with improved DBS outcomes during the 6-month period following surgery. Of the 154 patients who underwent DBS, 38 received leads in the STN (Right = 15, Left = 22, Bilateral = 1), 114 received leads in the GPi (Right = 44, Left = 67, Bilateral = 3), and 2 received leads in the Pedunculopontine nucleus. Because the DBS-CRS score did not differ between patients who received STN (mean = 2.33, *SD* = 0.82) versus GPi (mean = 2.52, *SD* = 0.91) DBS (Mann–Whitney *U* = 1,874.50, *Z* = −1.26, *p* = 0.209), we combined them into a single group for follow-up analyses.

Follow-up correlation and regression analyses focused on quality of life (PDQ-39) and mood, though additional motor and ADLs scores from the UPDRS were examined. Neuropsychological data was not available at this time interval. For these analyses, patients were excluded if they experienced an intra-operative adverse event (e.g., stroke, *N* = 1), post-operative infection (*N* = 6), had bilateral DBS surgery within the 6-month period (*N* = 34), or surgery not involving the STN or GPI (*N* = 2). An additional 16 participants were missing all scores from the PDQ-39. This resulted in a final follow-up sample of 95 participants.

Results of bootstrapped hierarchical linear regressions, controlling for education, revealed that the DBS-CRS served as a significant predictor for three subscales from the PDQ-39: ADLs (β = 0.20, *p* = 0.047), Cognitive (β = 0.20, *p* = 0.047), and Communication (β = 0.25, *p* = 0.013). Thus, higher cognitive risk was associated with worse quality of life post-surgery in three PDQ-39 domains. In additional correlational analyses (Spearman rho), higher cognitive risk (DBS-CRS) was significantly associated with worse post-DBS motor symptoms [UPDRS-Part III (on medication): *r* = 0.23, *p* = 0.046], and tended to be associated with worse ADLs post-surgery [UPDRS-Part II: *r* = 0.207, *p* = 0.052]. Neither the BDI-II nor the AS demonstrated significant relationships with the DBS-CRS (*p*’s > 0.05).

## Discussion

Neuropsychologists’ clinical judgment, as summarized by the DBS-CRS tool, plays an important role in the University of Florida’s DBS Fast Track evaluation of whether a patient should proceed to DBS surgery. Cognitive concerns, either alone or in combination with other concerns (i.e., psychiatric, neurologic), were the most cited reasons for patients not proceeding to surgery and accounted for 57% of excluded cases. These findings align with a robust literature that cognitive concerns are the primary reason PD patients do not proceed to DBS surgery (Lopiano et al., 2002; Abboud et al., 2014). In our study, comparing patients who did and did not proceed to surgery revealed no differences in demographic factors, most clinical features, or mood/motivation. However, those who did not proceed to surgery had comparatively worse cognitive performance across the board—on the DBS-CRS, a dementia screener (DRS-2), and all neuropsychological domains except visuoperception—further supporting our study’s hypothesis that cognition serves as an important factor in choosing which candidates proceed to DBS surgery.

What aspects of the neuropsychological exam contributed to clinical decision-making regarding DBS-CRS ratings? The strongest contributors, based on regression analyses, were delayed memory and executive function, with a weaker but significant influence from language and visuoperception domains. Indeed, 77.2% of the variance in the DBS-CRS scores were accounted for by cognitive performance, in conjunction with education and mood scores. We suspect that the remaining variance (22.8%) could be explained by non-cognitive factors not assessed in this study such as other medical conditions, cardiovascular risk factors, and frailty. Because this information was collected from the neuropsychological interview and the medical record review, the data could have possibly affected clinicians’ judgment.

One question of interest was whether worse DBS-CRS ratings would primarily reflect overall cognitive decline or may also reflect an atypical neuropsychological profile (e.g., atypical PD). Based on patients with the highest DBS-CRS scores (4–5) having significantly worse, broad impairment across most domains, our data are more in alignment with the former view of overall cognitive decline. However, we cannot fully dismiss the importance of atypical profiles, as these profiles may raise concerns about other co-occurring disease entities. These cases tend to be rare during DBS screening at expert centers, but it is clinically salient and critically important to communicate to the interdisciplinary team when uncovered.

A critical test of the utility of the DBS-CRS is whether this summary index would be associated with future adverse behaviors following DBS surgery. To address this point, we focused on quality of life and mood, as these measures provided a more patient centric view of outcome, rather than on cognition. Cognition scores were not available over the 6-month post-DBS surgery period. Indeed, we found that better DBS-CRS scores were linked to better ADLs, Cognition, and Communication scores on the PDQ-39 post-surgery. Moreover, we found that worse cognitive risk (DBS-CRS) was associated with worse motor scores and a trend for worse ADLs on the UPDRS. There was no relationship with mood. At least one prior study has reported that those with impaired cognitive performance did not exhibit improvements in quality of life after STN DBS in the same manner as those without cognitive deficits (Witt et al., 2011). To our knowledge, however, the current study is the first to specifically document the predictive relationship between quantified *cognitive risk* pre-DBS surgery and aspects of quality of life following DBS surgery.

Overall, while DRS-CRS serves as a helpful communication tool to other clinicians, there are limitations to its use. It is not intended to serve as a substitute for a comprehensive neuropsychological evaluation, which typically describes neuropsychological strengths and weaknesses and provides detailed recommendations beyond DBS suitability. In certain cases, those with poor cognitive performance, may be deemed adequate candidates for humanitarian reasons to optimize clinical care. That said, the DBS-CRS rating is an important communication tool that serves as one factor amongst many when assessing DBS candidacy.

Our study had several limitations. First, the majority of DBS-CRS ratings were assigned by the same attending neuropsychologist in conjunction with fellows and trainees. Thus, the generalizability of our findings is uncertain because we do not know how individual clinical judgment skills would affect DBS-CRS score assignment. Thus, future research should examine the inter-rater reliability between different raters at various levels of training. Preliminary work by our group suggests convergence by the clinical team after 3–4 weeks of training (i.e., around 12–15 cases), though this observation will need to be verified in more controlled settings. Generalizability of the scale’s use would also be improved with a more racially heterogenous sample. Another potential limitation is that the current study evaluated cognitive performance using theoretically determined domain classifications. Future research could use empirically-derived composite scores to predict DBS-CRS to further evaluate which cognitive impairments best predict the scores. Finally, future work should expand to examine the DBS-CRS’ role in predicting cognitive functioning post-DBS surgery and break down DBS candidates by target, as well as incorporate bilateral cases, to better understand individual differences between different DBS treatment regiments.

Overall, the DBS-CRS in this single center cohort was useful in deciding which Fast Track candidates should move onto DBS surgery. The study revealed that cognitive performance involving delayed memory and executive functioning were the strongest predictors of the DBS-CRS. The score on the DBS-CRS predicted the post-DBS surgery quality of life, specifically the ADLs, Cognition, and Communication domains. Based the current study’s findings, we believe that that the DBS-CRS has the potential to improve the DBS screening process by providing a simple score to aid decision making in potential surgical candidates.

## Data Availability Statement

The datasets presented in this article are not readily available because “an inquiring party must submit a request to the UF INFORM database committee at the Norman Fixel Institute of Neurologic Diseases, and this request must be approved by the UF IRB.” Requests to access the datasets should be directed to “Chuck Jacobson, jacobson@neurology.ufl.edu.”

## Ethics Statement

The studies involving human participants were reviewed and approved by University of Florida Institutional Review Board. The patients/participants provided their written informed consent to participate in this study.

## Author Contributions

All authors listed have made a substantial, direct and intellectual contribution to the work, and approved it for publication.

## Conflict of Interest

DB has received research grants, as PI or MPI, from the NIH (NINDS, NIMH, NIA), Parkinson’s Foundation, the McKnight Research Foundation, and the UF Foundation. MO serves as a consultant for the Parkinson’s Foundation, and has received research grants from NIH, Parkinson’s Foundation, the Michael J. Fox Foundation, the Parkinson Alliance, Smallwood Foundation, the Bachmann-Strauss Foundation, the Tourette Syndrome Association, and the UF Foundation. MO has received royalties for publications with Demos, Manson, Amazon, Smashwords, Books4Patients, Perseus, Robert Rose, Oxford, and Cambridge (movement disorders books). MO is an associate editor for New England Journal of Medicine Journal Watch Neurology. MO has participated in CME and educational activities on movement disorders sponsored by the Academy for Healthcare Learning, PeerView, Prime, QuantiaMD, WebMD/Medscape, Medicus, MedNet, Einstein, MedNet, Henry Stewart, American Academy of Neurology, Movement Disorders Society and by Vanderbilt University. The institution and not MO receives grants from Medtronic, Abbvie, Boston Scientific, Abbott, and Allergan and the PI has no financial interest in these grants. MO has participated as a site PI and/or co-I for several NIH, foundation, and industry sponsored trials over the years but has not received honoraria. Research projects at the University of Florida receive device and drug donations. KF reports grants from NIH, and other funding from Donnellan/Einstein/Merz Chair, during this study; grants and non-financial support from Medtronic, grants from St. Jude, Functional Neuromodulation, and Boston Scientific, and grants and other funding from Neuropace. Additionally, KF has a patent US 8295935 B2 issued for a DBS cranial lead fixation device. The remaining authors declare that the research was conducted in the absence of any commercial or financial relationships that could be construed as a potential conflict of interest.
